# Influence of Bubble Deformation on the Signal Characteristics Generated Using an Optical Fiber Gas–liquid Two-Phase Flow Sensor

**DOI:** 10.3390/s21217338

**Published:** 2021-11-04

**Authors:** Yu Ma, Yangrui Zhang, Song Li, Weimin Sun, Elfed Lewis

**Affiliations:** 1 Key Lab of In-Fiber Integrated Optics, Ministry Education of China, Harbin Engineering University, Harbin 150001, China; vicky_mic@hrbeu.edu.cn (Y.M.); s320250030@hrbeu.edu.cn (Y.Z.); lisong@hrbeu.edu.cn (S.L.); 2 Optical Fibre Sensors Research Centre, University of Limerick, V94 T9PX Limerick, Ireland

**Keywords:** optical fiber sensor, two-phase flow measurement, bubble deformation measurement

## Abstract

The use of optical fiber probe in two-phase flow measurements is very frequently encountered, especially in the applications of chemical engineering and petroleum industries. In this work, the influence of bubble piercing signals caused by bubble deformation is studied experimentally using a laboratory-prepared wedge-shaped fiber probe in a lab-scale gas–liquid flow generator. A three-dimensional simulation model is established to study the influence of bubble deformation on the piercing signals. A theoretical analysis of the characteristics of the pre-signal influenced by the bubble deformations is undertaken for a wide range of different modeled bubble shapes. Combining the experimental and simulation results, a promising analytical method to estimate the bubble shapes by analyzing the characteristics of pre-signals is proposed. The results of this investigation demonstrate that it is possible to estimate the bubble shapes before the fiber probe contacts the bubble surface. The method developed in this investigation is therefore highly promising for reducing errors caused by deformation during the probe piercing process.

## 1. Introduction

Two-phase flow environment widely exists in chemical, petrochemical and biochemical industries. The accurate analysis of the movement of bubbles in liquid has become a significant challenge when making measurements in gas–liquid two-phase flows [1,2,3]. The bubble motion in liquids usually manifests as a complex transient and is a dynamic process that can be characterized as a highly non-steady-state process. The overall dynamic process of rising bubbles in a fluid is often accompanied by complex deformation of the bubbles and this is further complicated by the existence of partial media interfaces in the dynamic bubble evolution. Thus, being able to accurately characterize the bubbles’ deformation, sizes and distributions varying with the liquid velocities, flow geometries, and local parameter information has become increasingly important in many natural and industrial applications [4,5,6]. Serious complications can be encountered when investigating bubble properties in gas–liquid flows, and hence non-invasive techniques have been constrained to determining only the local hydrodynamic parameters. Since Miller and Mitchie [7] put forward the principle for a fiber optic sensing technique as an invasive measurement method, this technique has become the preferred choice of a large number of practitioners in gas–liquid two-phase flow applications [8,9,10].

The use of optical fiber probes for the measurement of bubble velocity, bubble size distribution and bubble void fraction has become increasingly widespread and popular for use in two-phase flow detection [11,12,13,14]. However, as an invasive measurement method, the fiber probe brings potential deformation to the partial media intersection through the process of bubble piercing. In view of this issue, many researchers have conducted extensive experimental and simulation-based research for characterizing the deformation of the media interface in the process of the dynamics and statics of piercing by a cylindrical fiber probe [15,16,17,18]. Furthermore, Sakamoto et al. [19] utilized a computational method to investigate the influence of the deformation of the partial gas–liquid interface caused by the fiber probe on the bubble piercing signals. The characteristics of different bubble piercing mechanisms were created by investigating a wide range of different piercing angles and positions in order to improve the bubble measurement accuracy [20,21]. However, their study related only to the transient signal characteristics, depending on the deformation of media interface at the moment of fiber piercing, which means the measurement results do not include a global view of the bubble characteristics, i.e., the times immediately prior to the probe contacting the bubble surface. As a result, it would not be sufficiently accurate for applications for determining bubble shapes, gas holdups, bubble rise velocities, and chord lengths measurements. The influence of bubble shapes on the measurement results, pre-signal has not been analyzed theoretically and has therefore been largely overlooked in the recent literature.

In the study reported in this article, the experimentally observed pre-signal characteristics are established using a fully integrated wedge-shaped fiber probe for piercing different shapes of bubbles. The experimental results reported in this article show that when the fiber probe pierces a suborbicular air bubble and an approximate ellipse air bubble, the pre-signals are different. The measurements results show that the appearance of different pre-signals immediately prior to bubble piercing is strongly influenced by the bubble shape. However, in bubble flow measurements, the local interaction between fiber probes and gas–liquid interfaces is a transient phenomenon. Even if the fiber probe was fixed and relatively motionless, the uncertainties during the experiments introduced by the relatively random process of bubble evolution render it impossible to perform a quantitative analysis of the luminous intensity of the reflected light from the bubble surface for varying bubble shapes. Thus, further investigations were conducted using a ray tracing simulation method. A three-dimensional simulation model was established to study the influence of bubble deformation on the piercing signals by changing the radii ratio of bubble minor semi-axis, as well as the bubble aspect ratio. Based on an optical theoretical analysis of the simulation results, different characteristics of the pre-signals were determined by considering the bubble rear surface shapes which is a significant factor in the generation of the energy concentration area in front of the front bubble surface. In the authors’ previous work, the mechanism of energy concentration area appeared in front of the bubble surface before the bubble piercing process was discussed in detail [22]. This study further elaborates the effects of bubble shapes on the energy concentration area, and hence its influence on the pre-signals. Combining the experimental and simulation results, a promising analytical method to estimate the bubble shapes by analyzing the characteristics of pre-signals is formulated. The method will further provide an experimental and theoretical basis for the establishment of bubble movement in real industrial applications, such as wastewater treatment [23,24] and pharmaceutical engineering [25].

## 2. Methodology

### 2.1. Fiber Optic Measurement System

**Figure 1:**
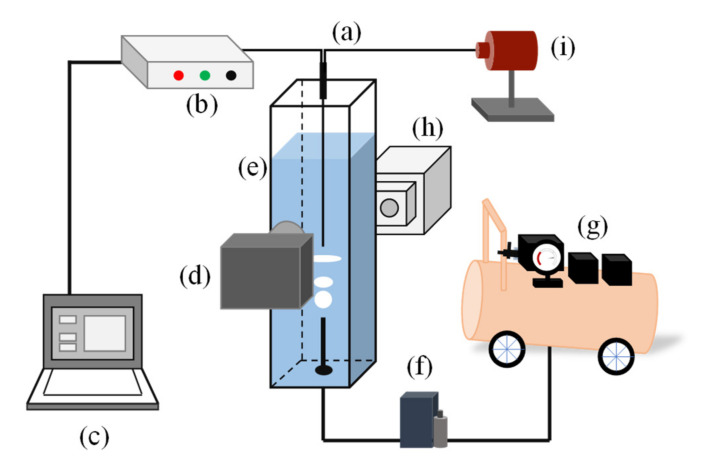
The experimental apparatus for bubble measurement: (**a**) optical fiber sensing system; (**b**) signal acquisition system; (**c**) PC; (**d**) DSLR camera; (**e**) rectangular vessel; (**f**) flow controller; (**g**) air compressor; (**h**) ultra-bright white light LED; (**i**) laser emitter.

The optical fiber sensing system employed in this study is the same as the one utilized in the author’s previous work [22] which describes all aspects of the optical fiber sensing system. All experiments of the investigation described in this article were undertaken in a purpose-built acrylic rectangular section chamber, with a stainless-steel capillary (inner diameter of 2 mm) located in the vessel at its bottom end. The gas was supplied using an oil-free air compressor (OTS-800-30L, Taizhou Outstanding Industry Additionally, Trade Co., Ltd., Wenling, China) and the gas flow rate was regulated at 0.2 L/min using a mass flow controller (ZF-10FD-LC, ZFKJ Technologies Co., Ltd., Beijing, China). The continuous gas flow was emitted from the capillary and detected by the fiber probe located above the capillary. A digital single lens reflex (DSLR) camera (Nikon D800, Tokyo, Japan) was used to capture the bubble piercing image; meanwhile, the reflected light was simultaneously received by the optical fiber sensing system. The reflected light signal was received using a distal photodiode detector (SFH250V, Infineon Technologies AG, Munich, Germany) and converted to an analog voltage signal by a photo-amplifier circuit. The output voltage signal was transferred via a data acquisition card (USB-1252A, SMACQ Technologies Co., Ltd., Beijing, China) to a laptop PC. The experimental apparatus is depicted in Figure 1.

### 2.2. Ray-Tracing Simulation Method

**Figure 2:**
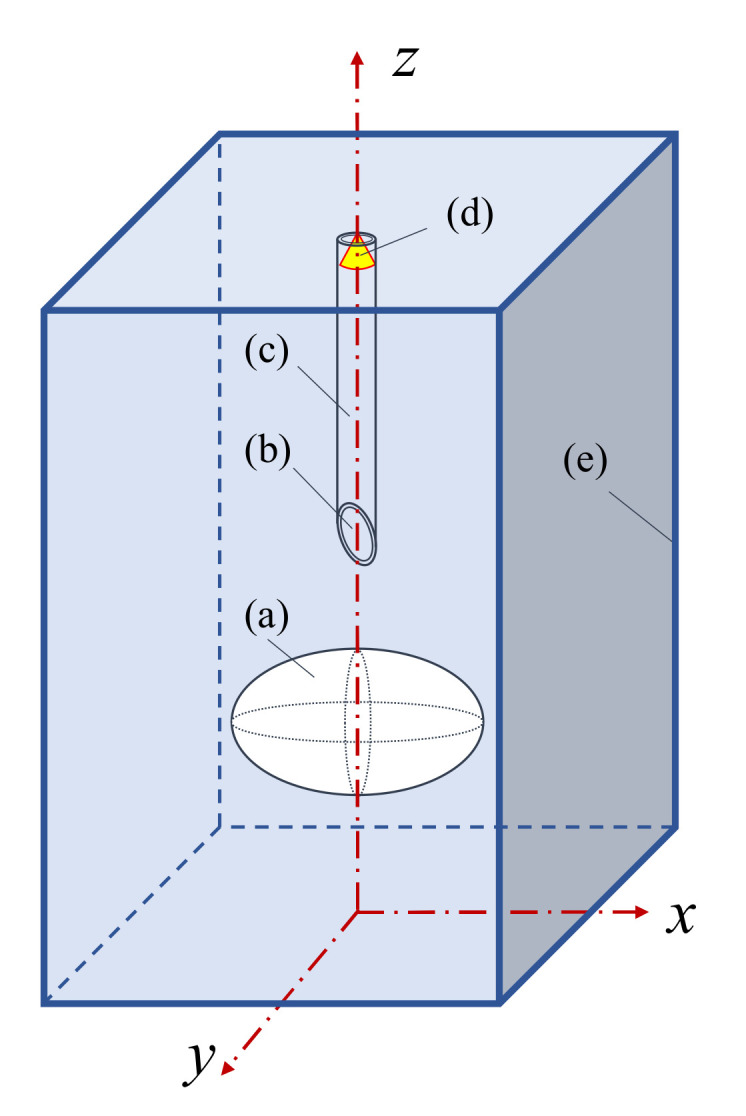
The configuration of optical model system: (**a**) Bubble (air); (**b**) Optical fiber core; (**c**) Optical fiber cladding; (**d**) Illuminant; (**e**) Water.

Due to the transient hydrodynamic process of the fiber probe bubble piercing, it was difficult to extract and analyze the pre-signal characteristics varying the bubble shapes purely from the experimental results. Thus, for the purpose of further analysis of the relationship between the bubble shapes and the measurement signal properties, a ray-tracing simulation method was implemented. A three-dimensional optical simulation model was established to simulate the process of different shapes of bubbles piercing in a cartesian coordinate system. The framework of the bubble piercing model is outlined in Figure 2.

The cube geometry (e) represents the water column. Inside the cube (e), an ellipsoid (a) represents the air bubble, the fiber probe comprises the fiber core (b) and fiber cladding (c), the illuminant (d) released from the end face of the fiber probe is represented as a conical beam of random rays with uniform density and distribution over the incident angle range from 0 to 20 deg along the direction of the negative *z*-axis.

The wedged angle of the fiber probe was set to 50 deg which is the same as the experimental probe utilized in this investigation. In this model, no hydrodynamic characteristics of the bubble surface were included in the computation, but instead relied on a quasi-static optical modeling of the fiber position with respect to the bubble axis. The position of the fiber probe apex relative to the bubble was controlled by shifting the coordinates of bubble frontal surface using a step length of 0.1 mm in the *z*-axis. The algorithm for the optical calculation was described in detail in a previous article by the authors of this article [22].

**Figure 3:**
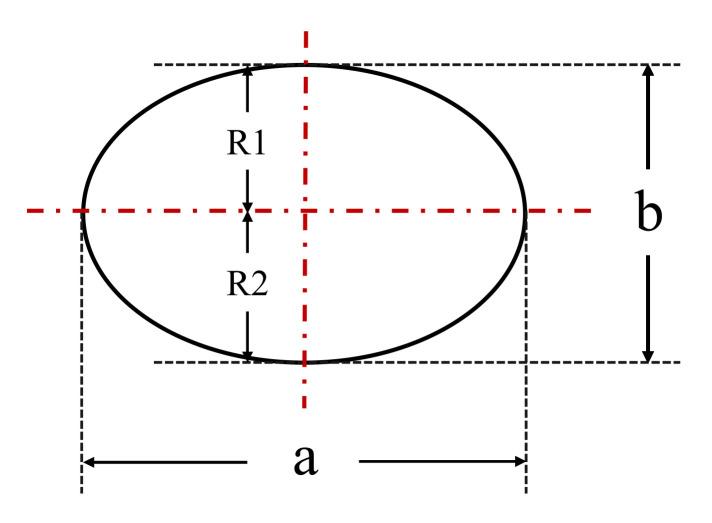
Axial plane of the modeled bubble.

A modeled bubble was created in order to offer different bubble shapes for the purpose of making a quantitative analysis of the pre-signal characteristics varying with the bubble shapes. The axial plane of the modeled bubble is shown in Figure 3. The parameters a and b represent the major axis and minor axis of the modeled bubble, respectively. R1 and R2 represent the upper and lower radii of bubble minor axis, respectively. The bubble aspect ratio was determined as *φ* = b/a., the value of *φ* changed by varying the upper and lower radii of bubble minor axis, which correspond to the different shapes visualized in the experimental investigations.

## 3. Results

### 3.1. Experimental Results

**Figure 4:**
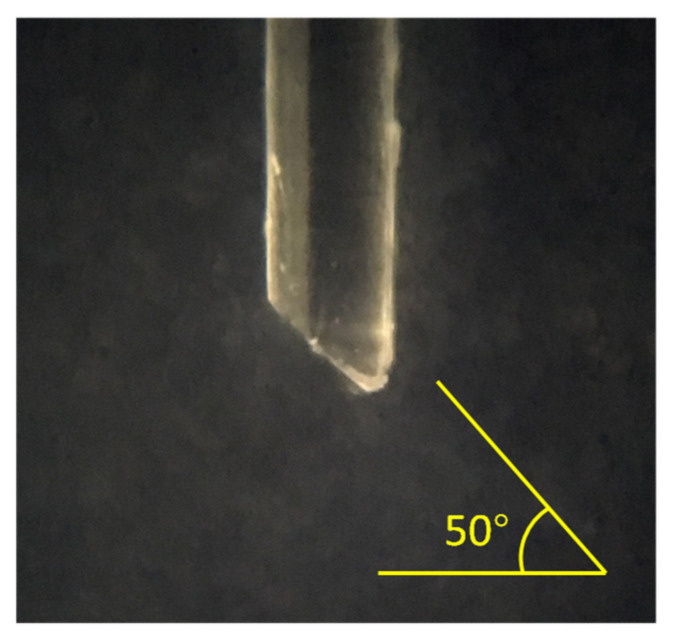
Photograph of a fabricated fiber sensing tip.

A wedge-shaped Poly-Methyl Methacrylate (PMMA) optical fiber tip with a wedge angle of approximately 50 degrees was utilized to detect the bubble flow signal under the conditions of different shapes of bubbles in the laboratory-scale gas–liquid flow generator described in Section 2.1. The wedged shape of the fiber probe was fabricated using the method described by Davenport et al. [26] and is the optimum angle for bubble piercing as determined in previous work by the authors of this article [22]. The microscope photograph of the tip of the fiber probe is shown as Figure 4.

**Figure 5:**
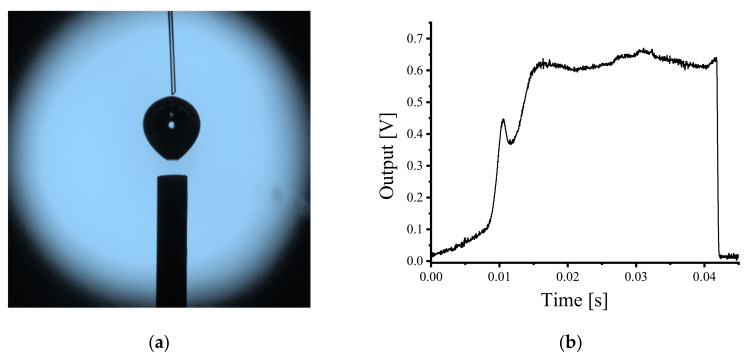
The fiber probe was placed approximately 10 mm above the air bleeder; (**a**) Captured bubble piercing image; (**b**) Time resolved measurement result.

In the visualization experiments, the bubble deformation trend was observed and ranged from a near perfect sphere to oblate spheroid from the instant the bubbles escape the air bleeder and rise in the water. The fiber probe was located at different locations above the exhaust capillary in order to obtain the measurement signals of different shaped bubbles. The experiment was undertaken in a homogeneous continuous bubble flow where the fiber probe was placed approximately 10 mm above the stainless capillary as previously described (Figure 1); Figure 5a shows an image of the bubble captured at the point of being pierced by the fiber probe; Figure 5b presents the time resolved output voltage signal for the fiber probe piercing a single bubble. In Figure 5a, it is clear that the emitted bubble was not deformed before the fiber probe contacted the bubble surface, and the bubble shape approximates to a sphere. The measurement result in Figure 5b shows a typical single-spike pre-signal appearing before the main square wave signal, which usually occurred at the moment of the fiber probe piercing the bubble frontal surface.

**Figure 6:**
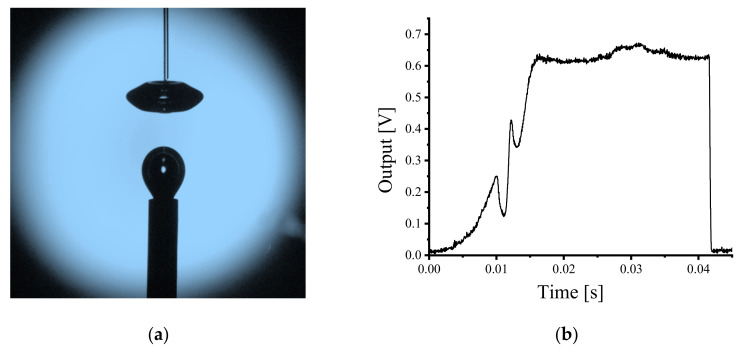
The fiber probe was located approximately 15 mm above the air bleeder; (**a**) Captured bubble piercing image; (**b**) Time resolved measurement result.

When the fiber probe was located at a higher position, being 15 mm above the air bleeder, there are clear differences in the time resolved experimental results. The captured bubble image is shown in Figure 6a. It is clear that after a period of rising in the water, the bubble shape approximates to an oblate spheroid just prior to being pierced by the fiber probe. The time resolved measurement result is shown in Figure 6b. Two spike signals appeared before the main pulse of the signal which together form the pre-signal. The first spike signal in Figure 6b is not as sharp as the second spike signal (typical single-spike pre-signal). Additionally, there exists an obvious time gap between the first spike signal and the rest of the signal curve, which indicates that the first spike signal occurs before the fiber probe touched the bubble frontal surface.

Work reported previously by the authors of this article demonstrated that the appearance of the pre-signal is related to the reflected light generated at the air–liquid surface (front bubble surface) [22]. Thus, the differences in the pre-signals between the two experimental results shown in Figure 5 and Figure 6 are likely related to the bubble deformations. For investigating the relationship between the characteristics of the pre-signal and bubble shapes, further studies of different shapes of bubbles piercing processes were undertaken using the computational method outlined in Section 3.2.

### 3.2. Simulation Piercing Processes for Different Bubble Shapes

The geometry and optical parameters of the modeled wedged fiber tip were the same as the experimental fiber probe used in this investigation. The horizontal axis of the simulation graphs represents the bubble frontal surface position relative to the fiber tip (corresponding to elapsed time), and the vertical axis corresponds to the calculated reflected optical output power signal from the fiber probe. The position that bubble frontal surface is just in contact with the fiber probe apex corresponds to the origin of the coordinate system.

**Figure 7:**
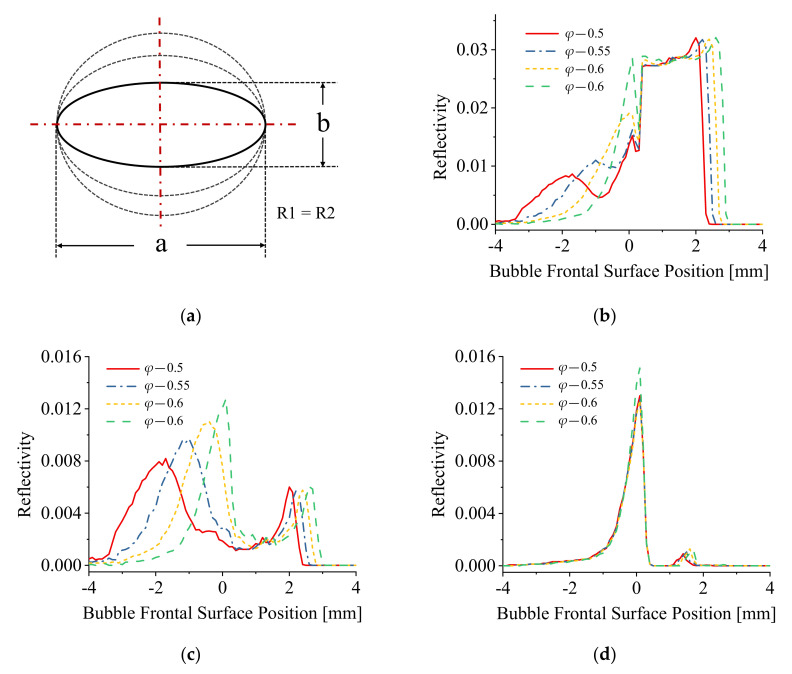
(**a**) The schematic diagram of bubble shape change; (**b**) The simulation results of piercing different shapes of bubbles; (**c**) The reflection signal formed on the rear surface of bubble; (**d**) The reflection signal formed on the frontal surface of bubble.

Figure 7 represents the geometry of the bubble used for the simulation (Figure 7a) and the results of the simulations are included in Figure 7b–d. In the case of the bubble geometry represented in Figure 7a, the modeled bubble shapes were changed by varying the value of bubble minor axis, for which the value of *φ* changed correspondingly. R1 and R2 were set equal. The graphs of Figure 7b represent the simulated reflection signals of a single bubble piercing for different values of *φ*. When the bubble shape was approximated to an oblate spheroid, a two peak pre-signal was generated which occurred just before the main bubble piercing rectangular pulse signal. With the bubble shape trending to a sphere, the first spike peak of pre-signal disappeared. These results are consistent with the experimental results presented in Section 3.1.

The variation of the pre-signal varying with the value of *φ* was investigated, including the reflection signals generated from the bubble rear and frontal surface, and the results of this simulation are presented in Figure 7c and Figure 7d, respectively. When the bubble shapes approximate to an oblate spheroid, the first spike peak signal is clearly evident. With the value of *φ* increasing, the bubble shapes approximate to a sphere, the fist spike peak signal approaches the second spike peak signal till the two spike peak signals superimpose, which makes the one-spike-peak pre-signal (as the experimental results in Figure 5). Changing the value of *φ* has a significant influence on the reflection signals generated on the bubble rear surface as shown in Figure 7c. However, the reflection signals generated on the bubble frontal surface were largely independent of the *φ* values. From the results in Figure 7, the characteristics of pre-signals were mostly dependent on the reflection signals generated on the bubble rear surface.

**Figure 8:**
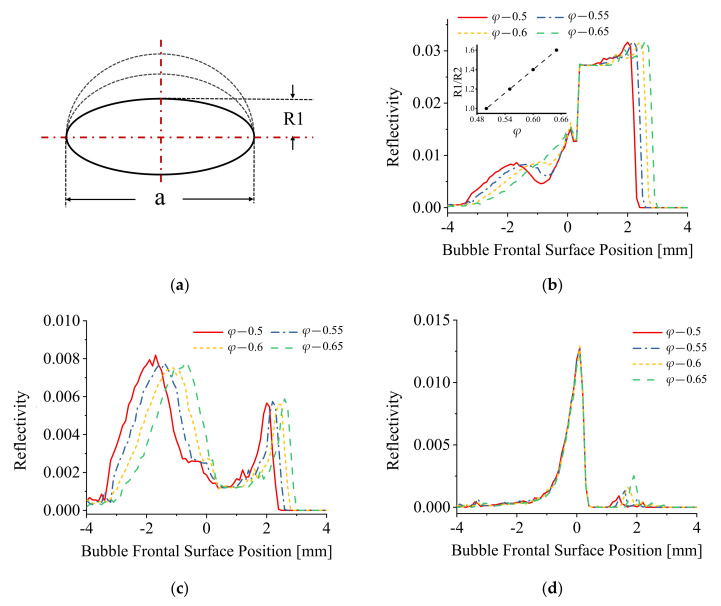
(**a**) The schematic diagram of bubble shape change; (**b**) The simulation results of piercing different shapes of bubbles (with only bubble frontal surface changing); (**c**) The reflection signal formed on the rear surface of bubble; (**d**) The reflection signal formed on the frontal surface of bubble.

**Figure 9:**
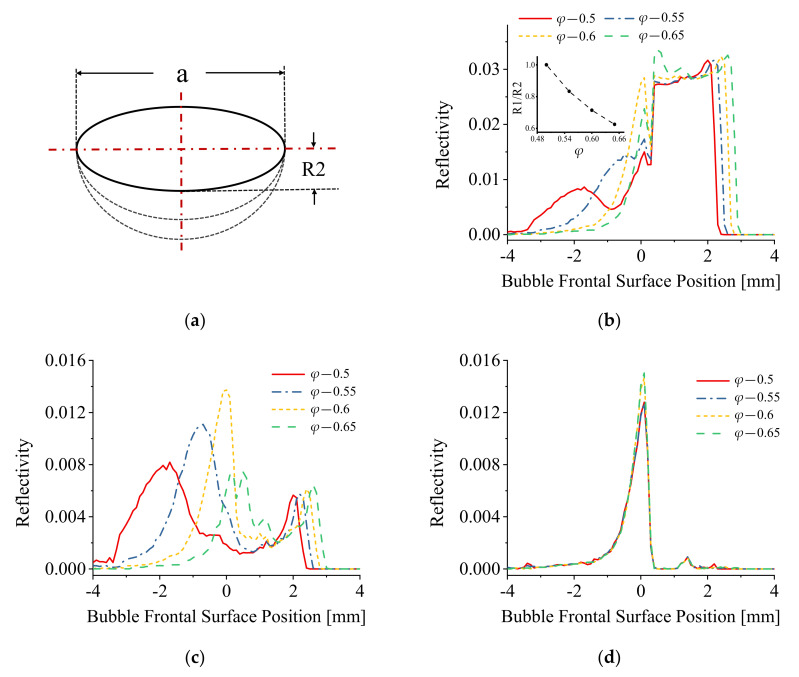
(**a**) The schematic diagram of bubble shape change; (**b**) The simulation results of piercing different shapes of bubbles (with only bubble rear surface changing); (**c**) The reflection signal formed on the rear surface of bubble; (**d**) The reflection signal formed on the frontal surface of bubble.

The detailed bubble deformation effect on the reflection signals generated on both the bubble rear and frontal surface required further computational analyses and these were conducted by separately changing the value of the upper and lower radii of bubble minor axis, R1 and R2. The simulation of the single bubble piercing at different *φ* values and different bubble radii ratio (R1/R2) are presented in Figure 8 and Figure 9.

Figure 8 shows the simulation results with only R1 being varied (R2 is fixed). With the increase in R1, the value of bubble aspect ratio *φ* increased correspondingly and the results are consistent with those obtained in Figure 7.

Figure 9 shows the computational results with R2 changing only. With the increase in R2, the bubble aspect ratio *φ* values increased correspondingly and are consistent with those of Figure 7 and Figure 8.

The results in Figure 7b, Figure 8b and Figure 9b indicate that when the bubble shape was approximated to an oblate spheroid, the pre-signal comprised two spike peaks; when the bubble shape trended to a sphere, the first spike peak of pre-signal disappeared. A further comparison of the signals generated on the bubble rear and frontal surface shows that the bubble deformation has a significant influence on the reflection signals generated on the bubble rear surface as shown in Figure 7c, Figure 8c and Figure 9c. Conversely, the reflection signals generated on the bubble frontal surface were almost independent of the bubble deformation as shown in the simulation curves in Figure 7d, Figure 8d and Figure 9d.

## 4. Discussion

It can be assumed that the modeled bubble in this simulation acts as a non-standard convex lens, the energy concentration area acts as an equivalent focus point, and the optic fiber probe acts as a moving point light source, the light emitted from the fiber tip is approximated as directional light. When the fiber probe was located at the equivalent focus point of the bubble (which was explained as the energy concentration area in the author’s previous work [22]), the fiber probe receives the maximum energy of the reflection signal generated from the bubble surface. Additionally, the deformation of bubble impacts the distance between the focus and bubble frontal surface, and hence the intensity of the light signal (energy) received in this area. From the results of Figure 7c, Figure 8c and Figure 9c, it can be concluded that the existence of the first peak of pre-signal was mainly determined by the extent of the equivalent focus point which is mainly generated by the bubble rear surface.

The appearance of the second peak of the pre-signal generated from the bubble fontal surface can be explained using the standard theory of light ray reflection [27,28]. As shown in Figure 7d, Figure 8d and Figure 9d, the reflection signals generated on the bubble frontal surface are largely independent of bubble deformation. When the modeled bubble approached the fiber probe, the reflection signals amplitude generally increased and reached the maximum value when the fiber probe touched the bubble frontal surface. After the fiber probe pierced the bubble, the reflection signal decreased. These observed phenomena demonstrated that when the fiber probe approached the bubble frontal surface, only regular reflection occurred, and no energy concentration effect was involved in this process.

The experimental and simulation results combined indicate that when the bubble shapes trend to an oblate spheroid, the focal length of the equivalent focus point increased, and the first spike peak appears. Conversely, when the bubble shapes approximate to a sphere, the focal length of the equivalent focus point decreased, and the two spike peak signals superimpose which manifests as a single spike peak pre-signal. The discovery of this phenomenon and its explanation provides a highly promising analytical method to distinguish several different bubble shapes through obtaining the measured time resolved characteristics of the signals. In particular, by analyzing the peaks of pre-signal, the basic bubble shape before the bubble piercing can be determined, offering a promising improvement in measurement accuracy in both industrial and general chemical application fields.

## 5. Conclusions

A wedge shaped PMMA optical fiber tip with a wedge angle of approximately 50 degrees was utilized to experimentally measure the time resolved bubble flow for a wide range of different bubble shapes in a laboratory-scale gas–liquid flow generator. In the visualization experiments, the bubble deformation trend was observed to evolve from near spherical to an oblate spheroid following their generation from the air bleeder and subsequent rise through the water column. Different pre-signal characteristics were observed in the bubble flow measurement when the fiber probe was placed at different locations above the exhaust capillary. In order to investigate the influence of bubble deformation on the characteristics of the pre-signal and discover the regular connection between the bubble shapes and the measurement signal, a ray-tracing based three-dimensional model was established to simulate the piercing process for different bubble shapes. Assuming the modeled bubble in this simulation acts as a non-standard convex lens, the simulation showed that changing shapes caused the focal distance of the equivalent focus point, which in turn leads to the changing of the maximum value and position of appearance of the peak caused by the bubble’s rear surface. The experimental and simulation results both indicate that when the bubble shapes tend to an oblate spheroid, the focal length of the equivalent focus point increased, and the first spike peak appears. Conversely, when the bubble shapes approximate to a sphere, the focal length of the equivalent focus point decreased and the two spike peak signals superimpose, which accounts for the appearance of the one-spike-peak pre-signal. Using this analytical method, it is possible to assess the bubble roundness in two-phase flow measurement, which is conducive to reducing the errors potentially caused by deformation by the piercing fiber probe.
